# Improving Patient behavioral Consent through Different Service Quality Dimensions: Assessing the Mediating Role of Patient Satisfaction

**DOI:** 10.3390/ijerph16234736

**Published:** 2019-11-27

**Authors:** Arif Jameel, Muhammad Asif, Abid Hussain, Jinsoo Hwang, Mussawar Hussain Bukhari, Sidra Mubeen, Insin Kim

**Affiliations:** 1 School of Public Affairs, Zijingang Campus, Zhejiang University, Hangzhou 310058, China or or or; 2 The College of Hospitality and Tourism Management, Sejong University, 98 Gunja-Dong, Gwanjin-Gu, Seoul 143-747, Korea; 3 Department of Political science, The Islamia University of Bahawalpur, Bahawalpur 63100, Pakistan; 4 Government College University, Faisalabad 38000, Pakistan; sidratarar@hotmail.com; 5 Department of Tourism and Convention, Pusan National University, Busan 43241, Korea; sini0721@naver.com

**Keywords:** service quality, patient behavioral consent, patient satisfaction, SEM, public sector hospitals, Pakistan

## Abstract

This study aimed to examine the impact of the five-dimensional health care service quality (SQ) on patient behavioral consent (PBC). This study further explored the mediating role of patient satisfaction (PS) on the SQ–PBC relationship. A survey questionnaire was used to collect the data from public sector hospitals situated in Bahawalpur division, Punjab, Pakistan. We used confirmatory factor analysis (CFA) and structural equation modeling (SEM) to test the hypotheses. This study found positive and significant relationships between SQ and PBC, SQ and PS, and PS and PBC. Our results further revealed that PS partially mediates the relationship between SQ and PBC. Our study offers a comprehensive theoretical framework of several service quality attributes (SQs) affecting patient behavioral consent (PBC) and patient satisfaction (PS) in health care institutions. Testing these above relationships via a mediation approach is novel and contributed to the current study on service quality.

## 1. Introduction

The measurement of service quality has been the concern of numerous research scientists and specialists. Grönroos [1] defined service quality (SQ) as an evaluation procedure in which the customer links his or her services prospects with perceptions. Thus, health care service quality measurement is becoming an important factor and should be addressed from the patient’s perspective because patients provide valid and unique information about the care they received [2]. Grönroos [1] suggested dual aspects of service quality. The first is technical, which denotes the main service delivery or service outcome, including provider competency as clinicians provide patient care. From the perspective of health care, these involve physician and nursing staff abilities and medical consequences. The second is practical care, which denotes service delivery procedures or the means through which the consumer obtains the services. In health care, these signify relational care and the socio-psychological associations between patients and providers. This includes provider–patient interactional quality (PPIQ), courtesy and the warmth that providers expressed to the patients. There are few studies that develop and test comprehensive models for capturing causality between various constructs [3].

**Figure 1:**
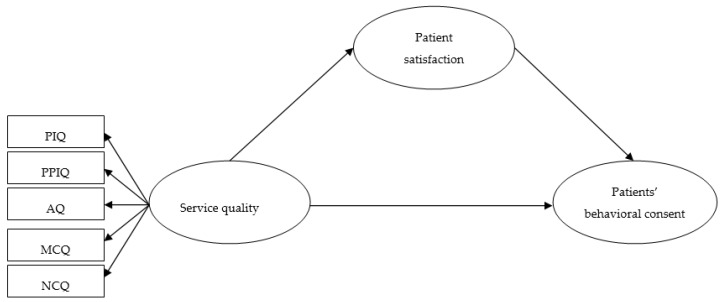
Proposed hypothesized model. This is the conceptual model of our study. Note, PIQ: physical infrastructure quality; PPIQ: provider–patient interactional quality; AQ: administrative quality; MCQ: medical care quality; NCQ: nursing care quality.

The principal objective of this research is to expand the growing health care quality research and the aforementioned aftermath in an emerging state—a Pakistani government sector health care institute. Pakistanis depend on government health care institutes, which offer somewhat free-of-charge services. Hence, the need to emphasize hospital service quality has become more relevant. Assessing service quality as observed by patients is one stage to advance government hospital service quality. This study’s specific objectives were to: (1) assess health care service quality using a five-dimensional model and (2) examine the influence of SQ on patient satisfaction (PS) and propose service quality enhancements for government sector hospitals. We established an alternate technique for assessing health care service quality in a greater context, where SQ, PS and patient behavioral consent (PBC) are the principal variables in our proposed theoretical model (Figure 1). Testing this five-dimensional model via the mediating influence of the patient satisfaction approach is novel and has not been explored previously. The theoretical model was devised from health care and psychology literature. Further, we recognize provider features and connect them to patient satisfaction and patient behavioral consent.

## 2. Theory and Hypotheses

### 2.1. Service Quality (SQ) 

Parasuraman, et al. [4], by means of the Service Quality (SERVQUAL) scale, presented significant contributions to understanding and assessing service quality. Thus, based on SERVQUAL, we considered the five-dimensional service quality construct. Research to determine service quality has commenced from a health care perspective in various states. In their work to assess health care service quality, Aagja and Garg [5] and Chahal and Kumari [6] recognized some aspects including admittance, medical and overall service, discharge, societal responsibility, physical atmosphere, communication and consequence as the most important. Hussain, et al. [7] advocates that relational facets, patient learning, price, technical facets, results, admittance times, services and societal support as service quality aspects in the setting of Pakistan. Others suggest a number of health care service quality dimensions, for instance, physical infrastructure, communication, experienced team, clinical and decision-making procedures, protection indicators, medical and nursing care quality (NCQ) and the services suppliers’ responsibilities [6,8].

The literature shows that SQ has different dimensions in different studies. Thus, further testing and validation are required before we accept any construct as dimensions underlying SQ [9]. Moreover, SQ is context-specific; studies performed in other contexts cannot be generalized [10]. In this research, founded on the literature, the physical infrastructure quality, provider–patient interactional quality (PPIQ), administrative quality (AQ), medical and nursing care quality (NCQ) constructs were proposed together as five-dimensional service quality.

### 2.2. Physical Infrastructure Quality (PIQ)

Health care services are dependent on PIQ to advance consumer experience. Healthcare services are high in credence qualities as such PIQ, which provides a cue for patients’ SQ perceptions [11]. Usually, in health care institutes, PIQ, for instance, physical services, equipment, employees and printed materials must appear good to create positive impressions and to effect promising patient opinions [7,12]. Research demonstrates that the association between physical infrastructure quality and service quality is substantial and positive [6,10].

### 2.3. Provider–Patient Interactional Quality (PPIQ)

The interfaces that occur between consumers and service staff during service delivery frequently influence service quality [13,14]. Health care services are imperceptible in nature and frequently compel patient participation in the cure procedure. In the procedure, patients also want information regarding their health status and outcomes, because absent information might affect the healing process. This condition adds to the close connections between the patient and the medical team. Hence, health care services highlight communications between patients and health care suppliers [15]. 

### 2.4. Administrative Quality (AQ)

The administrative quality helps essential services and concurrently inserts value for a consumer [16]. In hospitals, administrative quality contains admittance, “stay and discharge” procedures, clinical meetings and waiting time for a meeting. The administrative quality procedure is significant for safeguarding positive feedback [17]. Effectual administrative quality creates patients who appreciate health care institute services. Hence, accurately managed administrative quality is required to make the patient feel protected and have a pleasant experience in a hospital. Numerous research has argued that the association between administrative quality and service quality is substantial and constructive [8,10].

### 2.5. Medical Care Quality (MCQ)

Medical care quality is the technical aspect of health care quality that depicts the physicians’ performance, behaviors, activities, and manner regarding patient care. The construct evaluates patient experience regarding medical quality [8]. Its explanation is built on diagnostic correctness and processes or conformance to expert qualifications [18]. It denotes the physician’s capability, competence, and experience. Physicians also must demonstrate compassion, defined as courteous and professional communication conducive to engaging patient compliance and cooperation [12,19,20]. While patients put high importance on medical care quality, but how to assess the construct is usually not well understood. This service aspect is taken for granted by patients because they may not possess adequate related knowledge to evaluate the SQ offered by a doctor [21]. Research shows that the association between medical care quality and service quality is important and positive [10,22].

### 2.6. Nursing Care Quality (NCQ)

Nurses are in the majority in hospitals as the prime service provider and devote much time to patients in contrast to other medical workers [23]. Thus, NCQ is experienced and measured by patients [23,24], with the NCQ defined as the “delivery of safety care based on nursing standards, which eventuates in patient satisfaction”. Scholars confirm that nursing care quality is strongly and considerably correlated with service quality [10]. Nursing care quality is contemplated as the utmost essential element in client views. If the nursing staff are incapable of accomplishing their jobs, then health care service quality will not be attained. Patients gratified with nursing care quality create positive responses to nurses and further medical personnel.

### 2.7. Patient behavioral Consent (PBC)

In this research, patient behavioral consent refers to a precise patient behavior during and after receiving health care. In line with Hausman [25], patient behavioral consent denotes the level to which clients obey instructions and information. Thus, health care is dependent on patient support throughout service delivery to confirm operative services. Compliance with treatment is among the greatest health care problem for safeguarding operative cure, charge control, patient care and patient satisfaction [26]. Consequently, evidence demonstrates that sick individuals gratified with health care fulfill the treatment regime and have improved results [27,28].

### 2.8. Patient Satisfaction (PS)

SQ and PS are both connected but different variables, i.e., service quality is a rational variable, whereas patient satisfaction is a sentimental variable [29,30]. According to Zineldin [15], patient satisfaction is an emotional response, including indicators for satisfaction, with numerous health care quality aspects, for instance, technical, practical, physical infrastructure, communication, and environment. It reflects care quality and is frequently considered an important healthcare quality construct [31]. Scholars apply many elements to characterize patient satisfaction, for example, communication skills, workers’ competency, conduct and availability [32,33].

### 2.9. Service Quality, Patient Satisfaction, and Patient behavioral Consent

Much research has demonstrated that service quality and gratification with services have a varied influence on behavioral intents in the literature of marketing. Research endorses the idea that service quality is a precursor to consumer satisfaction [10,34]. From the perspective of health care, service quality has a constructive association with patients [35]. Research shows that service quality is circuitously linked to behavioral intents, with service satisfaction as a mediating variable [34,36]. Further, research also unearthed that service quality has a direct effect on behavioral intents [37]. In health care, scholars unearthed that the connection between service quality and patient behavioral consent is mediated by patient satisfaction; they also discovered that service quality directly impacts patient behavioral consent [26,38].

Therefore, there is a robust association between SQ, PS, and PBC in health care services. Consequently, we are interested in determining whether service quality has a direct effect on patient satisfaction and patient behavioral consent. We are also investigating whether patient satisfaction has a mediated influence on the association between service quality and patient behavioral consent.

**Hypothesis** **1** **(H1).** *Service quality has a significant and positive influence on patient satisfaction.*

**Hypothesis** **2** **(H2).** *Patient satisfaction has a significant and positive influence on patient behavioral consent.*

**Hypothesis** **3** **(H3).** *Service quality has a significant and positive influence on patient behavioral consent.*

**Hypothesis** **4** **(H4).** *Patient satisfaction mediates the relationship between service quality and patient behavioral consent.*

## 3. Methodology

### 3.1. Sampling and Data Collection

We measured causal relationships between the 5 dimensions of SQ, PS, and PBC in the health care sector. To obtain the most reliable health care quality estimates from hospital users and without population lists from which a random sample could not be drawn, we used the methods adopted by previous researchers; i.e., a population-based survey, especially stage-wise area sampling [39,40]. The respondents answered the questionnaire away from the hospital; i.e., they are not in contact with service providers (doctors and nurses), so are less affected by courtesy or gratitude bias [16,41]. These patients were hospitalized in the emergency room, adult medical ward, and surgical ward. Moreover, the population-based survey is exempted from the ethics committee review [42]. The population was defined as working public servants from government establishments in Bahawalpur Division of Punjab. Punjab is the largest province of Pakistan and Punjab Bahawalpur is the largest district as well as division of Punjab. Patients come here for treatment from other provinces too. Although it is a developing area, we chose this region for our study. The sample is considered a good theoretical population because respondents come from different government establishments and comprise various age groups, with different experience and different working cultures [43]. A total number of 400 respondents (government employees) were taken from 4 public sector hospitals situated in Bahawalpur division, Punjab, Pakistan and were admitted to hospital for at least two days to accumulate enough hospital experience [38]. Moreover, a minimum sample size of 200 is necessary to be used if the research analysis is conducted through structural equation modeling in order to generate valid fit measures [44,45]. The survey questionnaires were distributed to the respondents from 13 to 27 June 2019. Finally, 245 completed surveys were received and analyzed with a response rate of 61.25 percent. All eligible individuals were invited to participate in the survey and gave permission for the survey. 

### 3.2. Demographic Characteristics

The percentage of males was 69.8%, and the percentage of females was 30.2%. Overall, 38.7% of respondents were aged 25 to 34, whereas 13.88% were younger than 20 years. In regard to marital status, 80.82% were married and 19.18% were single. Overall. In regard to education, approximately 51.02% had a bachelor’s level of education, and 4.49% had primary school education. (See Table 1).

### 3.3. Survey Questionnaire

To invoke the constructs in our theoretical model, formerly proved scales were used and altered. Every item was evaluated using a “5-point Likert-scale, ranging from 1 (strongly disagree) to 5 (strongly agree)”. We also organized a pilot study with 30 recently discharged individuals to make sure that the question content, phrasing, order, design, questions complexity, commands, and Likert-scale were suitable. The pilot study feedback was employed to improve the survey beforehand it was used for data gathering. In all, 35 items were involved in this survey questionnaire.

### 3.4. Measures

Physical infrastructure quality (PIQ) was assessed using the 5 item scale developed by Duggirala, Rajendran and Anantharaman [8] and Arasli, et al. [46]. A sample item for PIQ includes “There were adequate numbers of bathrooms and toilets in the ward.” The value of α for the PIQ scale was 0.82. This study adopted the 5 items provider–patient interactional quality (PPIQ) scale proposed by Dagger, Sweeney and Johnson [10]. A sample item for PPIQ included “The staff at the clinic always listen to what I have to say.” The reliability of this scale was 0.89. AQ was assessed using the 5 item scale proposed by Duggirala, Rajendran and Anantharaman [8] and Dagger, Sweeney and Johnson [10]. A sample item for AQ was “The administration system of the clinic is excellent.” The reliability (α) for this scale was 0.87. We adopted a 5 item scale to measure MCQ which was developed by Saad Andaleeb and Millet [47]. A sample item for MCQ was “The doctor was willing to answer my questions.” The reliability (α) for this scale was 0.83. A 5 item NCQ scale was adopted in this study, which was developed by [10,47]. A sample item was “Nurses attended to me sincerely when needed.” The reliability (α) for the NCQ scale was 0.90. We adopted a 5 item scale to measure PS that was developed by Dagger, Sweeney and Johnson [10]. A sample item for PS was “I feel good about coming to this clinic for any treatment.” The reliability (α) for this scale was 0.91. A 5 item PBC scale was adopted in this study, which was developed by Lin and Hsieh [48]. The reliability (α) for PBC the scale was 0.86. 

### 3.5. Common Methods Variance (CMV)

We applied Herman’s single-factor test to investigate the possibility of a common method and variance bias [49]. Through “exploratory factor analysis (EFA)”, employing principal axis factors, we found all eigenvalues greater than 1 resulted in every item assessing first succession latent constructs. Our study results revealed that no factor appeared with a variance higher than 50% of the variance extracted. These results suggest that this study has no bias issue.

## 4. Results

We used Smart PLS [50] to assess our theoretical model parameters. We used nonparametric bootstrapping on 245 cases, with 10,000 bootstrapping samples to obtain the standard error estimates [51]. 

### 4.1. Descriptive Statistics

The statistics values of the mean, standard deviation, and correlations of every observed variable are demonstrated in Table 2. The values of mean ranged from 2.89 to 3.67, and the standard deviation values ranged from 0.56 to 0.99. Table 2 also shows that the correlations among all studied variables are positive and significant. Table 2 further shows the discriminant validity of every construct where the statistical values of “average variance extracted (AVE)” are above the inter-correlational values [52,53].

### 4.2. Measurement Model

In this article, the measurement model was evaluated on “Confirmatory Factor Analysis (CFA)” [54] and demonstrated in Table 3, and the standard factor loadings, Cronbach’s α, values of AVE, and the CR of each construct are exhibited. The alpha (α) coefficients for PIQ, PPIQ, AQ, MCQ, NCQ, PS and PBC are 0.82, 0.89, 0.87, 0.83, 0.90, 0.91 and 0.86 respectively. These αs are greater than the suggested value of 0.70 [55,56]. The standardized factor loadings ranged from 0.75 to 0.80 for PIQ, from 0.77 to 0.89 for PPIQ, from 0.71 to 0.88 for AQ, from 0.78 to 0.83 for MCQ, from 0.81 to 0.89 for NCQ, from 0.80 to 0.87 for PS and from 0.77 to 0.84 for PBC. All factor loadings are above 0.50 [55] and contribute significantly. The values of average variance explained (AVE) for PIQ, PPIQ, AQ, MCQ, NCQ, PS and PBC are 0.63, 0.74, 0.70, 0.66, 0.69, 0.75 and 0.67 respectively. These statistics offer CV, as each of them is greater than the suggested value of 0.50 [57,58]. The composite reliability (CR) for PIQ, PPIQ, AQ, MCQ, NCQ, PS and PBC ranged from 0.85 to 0.92 and is greater than the recommended value of 0.60 [59,60].

### 4.3. Structural Model and Hypotheses Testing

After examining the measurement model, the later phase involved developing a structural assessment model. The structural assessment model is comprised of the assessments of path coefficients for analyzing the hypotheses. To test the hypotheses, we used PLS-SEM, with a maximum-likelihood estimate. The correlation among each construct is shown in Table 2, and the regression coefficients (β) are illustrated in Table 4. H1 for our research is SQ has a significant and positive influence on PS. We found support (β = 0.47, *t* = 16.73, and *p* < 0.01) for hypothesis 1 from Table 4. H2 is PS has a significant and positive influence on patient behavioral consent. Table 4 provides the evidence for this hypothesis (β = 0.39, *t* = 12.98, and *p* < 0.01). H3 is SQ has a significant and positive influence on PBC, and we found supportive evidence (β = 0.44, *t* = 13.51, and *p* < 0.01) from Table 4. We employed the “bootstrapping technique” with a 10000 bootstrapping sampling to establish the significance levels of the path coefficient [51]. Table 4 shows the analysis of results and hypotheses. The empirical results provide sufficient evidence for supporting hypotheses 1–3, where the range of t-values is from 12.98 to 16.73, i.e., greater than 1.96 [61,62].

### 4.4. Mediating Effects and H4

Hypothesis 4, the mediating impact of the association between SQ and PBC, was assessed employing the procedure in [63]. The mediating effect criterion was previously found: firstly, H1, the independent variable (SQ), was significantly correlated with the mediating variable (patient satisfaction); second, H2, the mediating variable (patient satisfaction), significantly affected the principle (PBC), and lastly, H3, the direct association (in absence of the mediating variable) between SQ and PBC was significant [63]. All three constructs and all paths (Table 5) were investigated simultaneously, which ensures the superior results to other methods [63,64]. To form the mediating influence, the indirect effect (Table 5) must be significant. We applied the “non-parametric b5otstrapping” method to check the significance of the indirect effect influenced by the “PLS-SEM path modeling approach” [63] (Table 5).

From the analysis, the path from SQ to PS (β = 0.47; t = 14.55; p < 0.01) and the path from PS to PBC (β = 0.32; t = 9.16; p < 0.01) were significant. Introducing mediation reduces the coefficient value between SQ and PBC from β = 0.39 and t = 12.98 to β = 0.21 and t = 5.67. Therefore, the association between SQ and PBC is mediated by patient satisfaction [65]. The outcomes backed hypothesis 4. At that point, we estimated the significance level of the indirect effect (the product between coefficients of a path from SQ-PS and PS to PBC). The t value for the indirect effects was estimated over the technique recommended by [63]. The t statistic (Table 5) for the indirect effects was 4.03, which showed the mediating influence of patient satisfaction with significance at p < 0.01 and further backed hypothesis 4. This result shows that SQ had a substantial indirect influence (β = 0.15; t = 4.03; p < 0.01) on PBC, which was intensely mediated by patient satisfaction. Hence, we found the partial mediating effect of PS on the SQ–PBC relationship.

## 5. Discussion and Implications

We propose a research model to measure health care quality which encompasses infrastructure, interaction, AQ, MCQ and NCQ with PS and BC. This study’s theoretical implication lies in the significant association between SQ, PS, and BC. While our analysis supports the accepted view that SQ, PS, and PBC are critical constructs in health care quality [10,66], the findings refine and extend the literature in several ways. First, PS emerges as the dominant, significant and indirect PBC determinant. Second, owing to substantial indirect effects via PS and its significant direct impact, SQ has the strongest total influence on PBC. The result indicates that the PS indirect effect on the relationship between SQ and PBC is a better model than the direct effect, as it explains a higher variance percentage in PBC. 

The findings also indicate that patients evaluate healthcare quality at the HCM overall level; i.e., at cognition level (SQ), at affect level (PS) and conation level (PBC) in agreement with [2]. The results can be used by managers to redesign hospital service quality measurement and their health care quality strategies. Hospital managers need to have an appropriate quality model to guide service providers in their continuous quality improvement efforts. 

## 6. Limitations and Future Research Directions

Our research has several limitations Firstly, this research was based on quantitative findings; future research should be carried out by applying a qualitative or mixed-method approach for more interesting results. The assessment of the health care services by the patient is an individual procedure and employing a quantitative technique like a survey cannot imitate all patient judgments. Employing qualitative techniques in addition to the quantitative methodology in future research might offer a greater understanding of the association between the SQ, PS, and PBC. Second, the outcomes of this research have been acquired on the basis of research in Bahawalpur public sector hospitals and the health care service provided by private hospitals is by nature dissimilar to that of the public hospitals, and thus it is suggested that other studies are conducted in private health care institutes, with the intention of enhancing knowledge and information concerning the association between these two variables in the presence of a mediator. Third, due to limited time, we only conducted this research in one province. In the future, this research study can be expanded to other provinces or countries to generalize the findings of study. Fourth, we collected data from discharged patients; in future, data should be collected from hospitalized patients to examine the research model’s suitability for hospitalized patients. Information obtained from patients may not be free from subjectivity. Respondents might not have given truthful information about service quality and their responses might be different if they answered the questionnaire in hospital. Fifth, participants could not have provided correct facts about service quality, but their answers could be dissimilar if they responded to the survey in the hospitals. Finally, we used patient satisfaction as the potential mediator, adopting demographic variables (age, education, and gender) as the moderators could provide interesting results.

## 7. Conclusions

The health care quality model we established could be implemented to examine and advance the service provided to patients. We established in what way the study model might aid hospital personnel recognize health care aspects and features considered imperative by sick individuals. While the research model was established employing discharged individuals, it might be used by services suppliers offering greater involvement and better communication service. It is a prerequisite to apply the study model to patients in other health care services to further confirm its psychological features. Our study findings offer hospital administrators valuable insight into the health care services features that imitate patients perspectives on health service quality. Consequently, hospital administrators should place further importance on these features. These understandings can help administrators to shape decisions about patient well-being. These findings can also be utilized in quality enhancement endeavors, which is significant because of the influence of SQ on PS and patient behavioral consent. Our empirical findings suggest that in managing health care service quality, the entire five first-order constructs were significantly associated with SQ, PS, and PBC. Thus, managers could improve health care service quality perceptions by improving functional quality (i.e., PIQ, PPIQ, and AQ) and technical quality (i.e., MCQ and NCQ). They might base service quality enhancement strategies on the model’s parameters.

## Figures and Tables

**Table 1:** Demographic Characteristics.

Demographic Characteristics		Number (*n*)	%
Gender			
	Female	74	30.2
	Male	171	69.9
Age			
	18–24	34	13.9
	25–34	95	38.8
	35–44	76	31.0
	45–54	31	12.7
	≥55	9	3.7
Education			
	Primary	11	4.4
	Middle	23	9.3
	High school	19	7.8
	Intermediate/Diploma	48	19.6
	Bachelor	125	51.0
	Master	14	5.8
	Others	5	2.0
Marital status			
	Single	47	19.1
	Married	198	80.9

**Table 2:** Descriptive statistics and correlation among all variables.

	Mean	SD	PIQ	PPIQ	AQ	MCQ	NCQ	PS	PBC
PIQ	3.47	0.99	**(0.79)**						
PPIQ	2.89	0.56	0.26 **	**(0.86)**					
AQ	3.18	0.84	0.34 **	0.29 **	**(0.84)**				
MCQ	3.09	0.77	0.42 **	0.33 **	0.38 **	**(0.81)**			
NCQ	3.31	0.88	0.30 **	0.36 **	0.47 **	0.34 **	**(0.83)**		
PS	3.67	0.75	0.41 **	0.40 **	0.43 **	0.28 **	0.49 **	**(0.87)**	
PBC	3.14	0.64	0.29 **	0.27 **	0.30 **	0.36 **	0.41 **	0.32 **	**(0.82)**

Significance: ** *p* < 0.01. Bold values in parenthesis are the square root of average variance (AVE) showing discriminant validity. Note, SD: standard deviation; PIQ: physical infrastructure quality; PPIQ: provider–patient interactional quality; AQ: administrative quality; MCQ: medical care quality; NCQ: nursing care quality; PS: patient satisfaction; PBC: patient behavioral consent.

**Table 3:** Measurement model.

Construct	Factor Loadings	α	AVE	CR
**Physical infrastructure quality**	-	0.82	0.63	0.85
Toilets were kept clean.	0.77			
There were adequate numbers of bathrooms and toilets in the ward.	0.80			
The hospital was always neat and clean.	0.77			
All medicines were available at the hospital.	0.75			
All equipment was available at the hospital.	0.81			
**Provider-patient interactional quality**	-	0.89	0.74	0.91
The staff at the clinic always listen to what I have to say.	0.77			
The clinic’s staff treat me as an individual and not just a number.	0.83			
I feel the staff at the clinic understand my needs.	0.87			
The staff at the clinic are concerned about my well-being.	0.87			
I always get personalized attention from the staff at the clinic.	0.89			
**Administrative quality**	-	0.87	0.70	0.88
The administration system at the clinic is excellent.	0.76			
The administration at the clinic is of a high standard.	0.71			
I have confidence in the clinic’s administration system.	0.88			
The registration procedures at the clinic are efficient.	0.87			
The discharge procedures at the clinic are efficient.	0.83			
**Medical care quality**	-	0.83	0.66	0.87
I find it easy to discuss things with the staff at the clinic.	0.78			
The staff at the clinic explain things in a way that I can understand.	0.81			
The staff at the clinic are willing to answer my questions.	0.81			
I believe the staff at the clinic care about me.	0.80			
I always get personalized attention from the staff at the clinic.	0.83			
**Nursing care quality**	-	0.90	0.69	0.92
Nurses behaved well.	0.81			
I had confidence in the nurses.	0.84			
Nurses were available when needed.	0.82			
Nurses were experts.	0.89			
Services were available promptly.	0.84			
**Patient satisfaction**	-	0.91	0.75	0.90
My feelings towards the clinic are very positive.	0.80			
I feel good about coming to this clinic for my treatment.	0.85			
Overall, I am satisfied with the clinic and the service it provides.	0.84			
I feel satisfied that the results of my treatment are the best that can be achieved.	0.85			
The extent to which my treatment has produced the best possible outcome is satisfying.	0.87			
**Patient behavioral consent**	-	0.86	0.67	0.89
If I had to start treatment again, I would want to come to this clinic.	0.77			
I would highly recommend the clinic to other patients.	0.80			
I have said positive things about the clinic to my family and friends.	0.81			
I intend to continue having treatment, or any follow-up care I need, at this clinic.	0.80			
I have no desire to change clinics.	0.84			

**Table 4:** Path estimates (β) for testing hypotheses 1–3.

Hypotheses	Path	Standardized β	T	Sig.	Results
Hypothesis 1	SQ→PS	0.47	16.73	*p* < 0.01 (**)	Supported
Hypothesis 2	PS→PBC	0.39	12.98	*p* < 0.01 (**)	Supported
Hypothesis 3	SQ→PBC	0.44	13.51	*p* < 0.01 (**)	Supported

Note, SQ: service quality; PS: patient satisfaction; PBC: patient behavioral consent. Significance level: ** *p* < 0.01.

**Table 5:** Mediating Effects.

Path	β	t	LLCI	ULCI	Sig.
Direct effect					
SQ→PS	0.47	14.55	0.27	0.62	*p* < 0.01 (**)
PS→PBC	0.32	9.16	0.31	0.68	*p* < 0.01 (**)
SQ→PBC	0.21	5.67	0.17	0.33	*p* < 0.01 (**)
Indirect effect					
SQ→PS→PBC	0.15	4.03	0.24	0.52	*p* < 0.01 (**)

Note, SQ: service quality; PS: patient satisfaction; PBC: patient behavioral consent; LLCI: lower limit confidence interval; ULCI: upper limit confidence interval. Significance level: ** *p* < 0.01.
